# Cultural stress, family functioning, hazardous alcohol use, and mental health among Latin American parents in the United States: A latent profile analysis

**DOI:** 10.1371/journal.pone.0337543

**Published:** 2025-11-21

**Authors:** Andrea Lopez-Soto, Esmeralda Ramírez, Duyen H. Vo, Aigerim Alpysbekova, Seo Woo Lee, Maria Duque, Lawrence Watkins, Cory L. Cobb, Beyhan Ertanir, Alejandra Garcia Isaza, Evelyn Gualdron, Sumeyra Sahbaz, Collette Steed, Neel Devan Youts, Shriya Senapathi, Seth J. Schwartz, Pablo Montero-Zamora

**Affiliations:** 1 Department of Kinesiology and Health Education, University of Texas at Austin, Austin, Texas, United States of America; 2 School of Social Work, Boston College, Chestnut Hill, Massachusetts, United States of America; 3 School of Public Health, Texas A&M University, College Station, Texas, United States of America; 4 University of Basel, Basel, Switzerland; 5 Department of Educational Psychology, University of Texas at Austin, Austin, Texas, United States of America; 6 Department of Curriculum and Instruction, University of Texas at Austin, Austin, Texas, United States of America; Caleb University, NIGERIA

## Abstract

There is a limited understanding of how different subgroups of Latin American immigrant parents experience cultural stressors, as well as its impact on family dynamics, health behaviors, and mental health. The present study aimed to (1) identify latent cultural stress profiles among Latin American immigrant parents in the U.S. and (2) examine differences among these profiles concerning family intimacy, democratic parenting style, family conflict, hazardous alcohol use, and depressive and anxiety symptoms. Participants consisted of a sample of 1,351 parents (61.9% female; *M* age = 39.83, 62% first-generation; North America [61%], Central America and the Caribbean [21%], and South America [19%]) of children aged 8–16. We used latent profile analysis to identify subgroups of cultural stress, defined by perceived discrimination (PDS) and negative context of reception (NCR). Multinomial logistic regression was conducted to examine key correlates of profile membership. Five latent profiles were identified (1) Low PDS/NCR (22.2%), (2) Low PDS/Elevated NCR (14.8%), (3) Moderate PDS/NCR (18.7%), (4) Elevated PDS/NCR (33.5%), and (5) Highest PDS/NCR (10.8%). Compared with Profile 1 (Low PDS/NCR), parents in Profiles 2–5 generally reported lower family intimacy (RRR = 0.93–0.97). Parents in Profile 5 (Highest PDS/NCR) reported more family conflict (RRR = 1.13), hazardous alcohol use (RRR = 1.20), depressive symptoms (RRR = 1.31), and anxiety symptoms (RRR = 1.29), with markedly elevated depressive (RRR = 22.94) and anxiety symptomatology (RRR = 17.48) compared with Profile 1. Our findings suggest the presence of vulnerable subgroups due to cultural stress among Latin American parents in the United States. A better understanding of cultural stress patterns may improve current and future interventions tailored for Latin American families, addressing health disparities within this population.

## Introduction

The United States (US) is one of the leading destinations for immigrants, with an estimated 90.9 million, including both legal and undocumented individuals [1]. Among the various immigrant groups in the U.S., the Latin American population comprises approximately 60 million individuals [2]. Latin American immigrants (because Puerto Ricans are U.S. citizens at birth, they are not counted as Latin American immigrants in the US) primarily originate from Mexico, El Salvador, Guatemala, Honduras, Venezuela, and Cuba [3]. Historically, Mexicans have comprised the largest segment of this demographic, though recent years have seen a significant increase in the Venezuelan population [4]. Overall, Latin American individuals predominantly reside in U.S. territories and states such as Puerto Rico, New Mexico, California, Texas, Arizona, Nevada, and Florida [5].

Human migration is a long-standing phenomenon driven by different factors, including lack of economic opportunities (e.g., Venezuelan economic diaspora), conflict (e.g., migration due to gang violence in certain parts of Mexico and Central America Triangle [6,7]), and climate-related disasters (e.g., Hurricane Mitch in Central America [8]). These reasons for migrating are likely to lead to different health outcomes [9]. Immigration involves adapting to a new environment, and the effectiveness of this adaptation process can vary depending on several factors, such as age, sex, immigration status, and socioeconomic background [10]. The adaptation process can vary across different groups, sometimes leading to both some positive and some negative effects on individuals’ lives and health [11]. From a positive perspective, migration can lead to increased job opportunities, improved socioeconomic status, and enhanced well-being and quality of life [12]. From a negative perspective, individuals facing immigration-related stress—even under optimal socioeconomic conditions—may experience mental health challenges as they adapt to a new environment post migration [13]. The present study focuses primarily on these negative sequelae of migration.

Recent theories have introduced various frameworks to explore how post-migration circumstances affect immigrants and their families, such as cultural stress theory (CST [14]). Specifically, CST seeks to identify multiple chronic cultural stressors (e.g., perceived discrimination, negative context of reception, language stress) that affect individuals navigating new host environments [15]. Among the most widely studied cultural stressors are perceived discrimination and negative context of reception [16]. Perceived discrimination refers to direct negative and unfair treatment stemming from immigration-related experiences based on their immigrant status and ethnicity (e.g., prejudice, denigration, and violence) within the receiving environment [17]. Discrimination arises when immigrants perceive direct and inequitable treatment from different actors (e.g., peers, community members, and governmental authorities) due to their race, ethnicity, or national origin [18,19]. Negative context of reception refers to an individual’s perception of how the host society values their ethnic group [16]. Negative context of reception includes perceptions of unwelcomeness, limited opportunities, and insufficient social support within the new country due to an individual’s ethnic background. Whereas discrimination involves overt negative actions taken against a person or group, negative context of reception is more indirect and may not involve any specific behaviors or actions. For example, immigrants may not face language barriers or hurtful comments from host nationals, but they may nonetheless feel “shut out” of opportunities within the destination community [15,20].

According to CST, the stress associated with the experience of perceived discrimination and the negative context of reception can influence family dynamics, health behaviors, and mental health [14]. CST proposes that cultural stressors might impair family functioning-related constructs that have been established before migration (e.g., family intimacy, democratic parenting style), thereby likely influencing individual adjustment (e.g., coping strategies) and creating family conflict after migration [21,22]. Indeed, research has found that elevated cultural stress might negatively impact family cohesion and familial well-being among Latin Americans in the US [23,24].

Furthermore, cultural stressors may directly contribute to adverse behavioral and health outcomes. Specifically, among Latin American adolescents and adults in the U.S., such stressors have been associated with hazardous alcohol use and mental health problems (e.g., elevated depressive and anxiety symptoms [24–27]). Although research highlights the potential effects of cultural stress on Latin American individuals and their families, it has not yet addressed the heterogeneity *within* Latin Americans (e.g., Central Americans, South Americans, Caribbeans; first versus second generation immigrants). Specifically, there is a lack of understanding of how Latin American parents in the U.S. —and their subgroups based on country of origin and on nativity—experience and manifest differing levels of cultural stress in their lives. For instance, Montero-Zamora et al. [28] emphasize that acknowledging these differences within the Latin American community, rather than perceiving them as a singular group, is crucial for informing better preventive interventions designed to improve cultural stress coping strategies.

In seeking to understand the extent to which Latin American individuals experience cultural stress differently, a person-centered approach (PCA) provides an invaluable framework for identifying and understanding distinct stress-pattern subgroups. Unlike variable-centered analyses focusing on overarching sample trends, PCA identifies subgroups of participants defined by combinations of responses to the clustering or classifying indicators [29,30]. In other words, PCA seeks to identify the optimal number of subgroups that might be present in a sample and to define them based on their unique configurations across a predetermined set of variables [31].

To understand patterns of cultural stressors among immigrant Latin American populations, PCA offers insight into the heterogeneity of responses to cultural stressor-related scales (e.g., the perceived discrimination scale [32] and the negative context of reception scale [16] among Latin American parents in the United States. By facilitating a more nuanced categorization of cultural stress, PCA can enhance the comprehension of how various subgroups experience and manifest these challenges (e.g., harmful alcohol use [33]). Additionally, one form of PCA, Latent Profile Analysis (LPA), allows for examining distinct cultural stress patterns rather than treating cultural stress as a single latent construct (e.g., assuming cultural stressors co-occur to form a unified construct). The LPA approach allows for the possibility that discrimination and the negative context of reception may not always co-occur [15,34]. Based on the stress proliferation framework [35] and on CST, recent research by Ertanir et al. [36] on Hispanic adolescents highlights the differential impact of various cultural stressors—including perceived discrimination and negative context of reception—on both other cultural stressors and psychosocial outcomes. These findings suggest that examining these cultural stressors separately provides valuable insights into their unique and interrelated effects.

### The present study

Cultural stress poses a considerable challenge for Latin American immigrant families. However, there is a notable research gap regarding whether there are subgroups of Latin American individuals based on varying levels of cultural stressors—such as discrimination and negative reception contexts—among different types of Latin American immigrants (e.g., North American, Central American, or South American). Additionally, it is unclear how these cultural stressor level-based subgroups are associated with family dynamics, behavioral outcomes, and mental health outcomes. Therefore, in this study we aimed to (1) identify latent cultural stress profiles among Latin American immigrant parents based on varying levels of perceived discrimination and negative context of reception and (2) explore the differences among these profiles concerning family intimacy, democratic parenting style, family conflict, hazardous alcohol use, depressive symptoms, and anxiety symptoms.

## Method

### Sample

To conduct the present study, we used a sample of 1,351 Latin American parents (61.9% female, *M* age = 39.83 ± 7.11 years [range 18–61 years]) of children aged 8–16 was recruited. The sample’s birth origin was 61% from North America (i.e., United States: 39%; Mexico: 22%), 19% from South America (i.e., Colombia, Venezuela, Peru, Uruguay, Chile, and Argentina), 13% from the Caribbean (i.e., Cuba and the Dominican Republic), and 7% from Central America (i.e., Guatemala, El Salvador, Honduras, Nicaragua, Costa Rica, and Panama). Regarding household income–post-taxation–52% of participants indicated they live on less than $2,000 per month. Approximately 76% of participants indicated that they have a partner, 43% reported having earned a college degree or higher level of education, and 62% identified as first-generation immigrants (i.e., foreign-born).

### Procedures

Participants residing in the US during the study period (June 14, 2022 – February 28, 2023) completed an online web panel survey. Generally, web panels consist of individuals who have consented to participate in online surveys, typically recruited through social media, email invitations, and online advertisements. A non-probabilistic purposive sampling approach was used, with pre-established quotas based on family country of origin (i.e., Mexico vs. other Latin American countries) and immigration status (i.e., first- vs. second-generation). Participants had to be 18 or older, be parents of a child between 8 and 16, and self-identify as first- or second-generation Latin Americans (i.e., participants or their parents were from a Spanish-speaking country in the Americas). Parents who chose to participate in the study and met the eligibility criteria provided electronic informed consent (i.e., written consent) before completing an online survey, which lasted approximately 30–35 minutes. The survey addressed the following domains: (1) perceived cultural stressors (e.g., discrimination and negative context of reception), (2) family functioning (e.g., family intimacy, democratic parenting style, and family conflict), (3) hazardous alcohol use, and (4) mental health (e.g., depressive and anxiety symptoms). The web panel provider (i.e., Centiment), distributed incentives ($7 to $10) to participants upon survey completion. Survey data obtained from the web panel provider were shared as de-identified data with the corresponding author prior to analysis to ensure participant confidentiality and data security, and all data were stored in the secure cloud system of the corresponding author’s institution, which also approved all study procedures through its Institutional Review Board.

### Measures

#### Sociodemographic characteristics.

Participants provided information on their biological sex (i.e., male, female), age in years, and birth region, which included North America (United States or Mexico), South America (e.g., Venezuela), Central America (e.g., Honduras), and the Caribbean (e.g., Cuba). Notably, North American participants born in the U.S. were considered a homogeneous group because all individuals in our sample were adults who had lived their entire lives (~40 years) in the U.S., which might result in similar levels of acculturation. Participants also reported on household income (i.e., less than $2,000 or $2,000 and above), partnership status (i.e., partnered or not), educational attainment (i.e., less than a college degree or college degree and higher), and immigration status (i.e., first-generation–or foreign-born–, and second-generation–or US-born–).

#### Cultural stressors.

##### Perceived discrimination:

We used the seven-item scale by Phinney et al. [32], which assesses perceptions of discrimination. Sample items include (1) *‘How often do employers treat you unfairly or negatively because you are Latino?’* and (2) ‘*To what extent do you feel that you are not wanted in US society?’* Participants scored each item using a 5-point Likert scale ranging from 1 (*Never) to* 5 (*Almost always*). Cronbach’s alpha (α) for this study was.93.

##### Perceived negative context of reception:

We utilized the six-item scale created by Schwartz et al. [16]. Items demonstrate the perception that Latin American individuals face more limitations in opportunities than other migrant groups. The scale also measures perceptions regarding how the receiving new context does not accept or recognize Latin American immigrants. Examples of these items include (1) ‘*It is hard for me to do well at work because of where I am from,’* and (2) ‘*My family and I would be treated better if we were more like other immigrant groups*.’ Participants evaluated each statement using a 5-point Likert scale, ranging from 1 (*Strongly disagree) to* 5 (*Strongly agree)* (α = .87).

#### Family functioning.

The 30-item Intimacy, Conflict, and Parenting Style Family Functioning Scale (ICPS-FFS [37]) was used to measure family functioning. This scale comprises three sub-scales (1) family intimacy (12 items, e.g., ‘*People in our family help and support each other’*), (2) democratic parenting style (8 items, e.g., ‘*Each member of our family has a say in important family decisions’*), and (3) family conflict (10 items, e.g., ‘*Making decisions and plans is a problem for our family’*). Participants completed all sub-scales using a 6-point Likert scale, ranging from 1 (*Totally disagree*) to 6 (*Totally agree*). We calculated a total score for each family functioning sub-scale by summing the scores of its corresponding items. Higher scores indicated a greater level of the construct (family intimacy α = .91; democratic parenting style α = .78; family conflict α = .86).

#### Behavioral and mental health outcomes.

##### Hazardous alcohol use:

To evaluate hazardous alcohol use, we employed the 10-item Alcohol Use Disorders Identification Test (AUDIT [38]). This scale comprises (1) the measure of amount and frequency of alcohol intake (e.g., ‘*How often do you have a drink containing alcohol?’*), (2) frequency of alcohol-related behaviors (e.g., ‘*During the past year, how often have you failed to do what was normally expected of you because of drinking’*), and (3) alcohol-related problems (e.g., ‘*Have you or someone else been injured as a result of your drinking?’*). AUDIT scores were calculated by summing responses to all items (*M* = 7.22, *SD* = 8.40; α = .92).

##### Depressive symptoms:

To measure depressive symptoms, we employed the 10-item Center for Epidemiologic Studies Depression Scale Boston form (CES-D-B [39]). The questionnaire included eight negative items (e.g., ‘*I felt depressed’*) and two positive items (e.g., ‘*I was happy* and *I enjoyed life’*). Participants indicated how often they experienced depressive symptoms over the past two weeks using a 4-point Likert scale, ranging from 0 (*Rarely or none of the time)* to 3 (*Most or all of the time*). Positive items with inverted wording were reverse coded before calculating the summed responses. We computed total scores by adding the ten items, resulting in a range from 0 to 30, where higher scores signify more severe depressive symptoms. Items were summed to a total score (*M* = 9.26, *SD* = 6.67; α = .89). Elevated depressive symptoms were defined as a score ≥ 10 (40.64% [39]).

##### Anxiety symptoms:

We assessed anxiety symptoms using the 7-item Generalized Anxiety Disorder Scale [40]. Sample items included (1) ‘*Feeling nervous, anxious or tense’*, and (2) ‘*Being so restless it is difficult to sit calmly’*. Participants indicated how often they experienced anxiety symptoms over the past two weeks using a 4-point Likert scale, ranging from 0 (*Not at all*) to 3 (*Nearly every day*). Scores for each item were totaled, resulting in a cumulative score between 0 and 21 (*M* = 6.19, *SD* = 5.99; α = .93). Elevated anxiety symptoms were defined as a score ≥ 10 (27.50% [40]).

### Data analysis

Our data analysis followed a structured, multi-step approach. First, we conducted a LPA [41] to identify unobserved subgroups within the data. LPA, a statistical technique that estimates an individual’s probability of belonging to different latent profiles, was performed at the item level, using M*plus* 8 [42], with perceived discrimination (7 items) and negative context of reception (6 items) as continuous indicators. Missing data were handled using full information maximum likelihood (FIML), which estimates model parameters using all available data without imputing missing values, minimizing bias and preserving statistical power.

We used a multi-criteria decision process [43,44] to determine the optimal number of latent profiles. Specifically, we used the Bayesian Information Criteria (BIC), sample size-adjusted BIC (SSABIC), and the sample size-adjusted Lo-Mendell-Rubin likelihood ratio test. Model fit was evaluated based on entropy values of at least.80, lower BIC and SSABIC values, and statistically significant (*p* < .05) Lo-Mendell-Rubin likelihood ratio test (LMR-LRT) and bootstrap likelihood ratio test (BLRT) results.

Second, after identifying cultural stress latent profiles through LPA, we assigned parents to each profile based on their most likely profile membership. Third, we characterized each profile according to its standing in terms of sociodemographic factors and key study variables, including family intimacy, democratic parenting style, family conflict, hazardous alcohol use, and depressive and anxiety symptoms. Finally, we conducted a series of multinomial logistic regressions [45] to examine associations between cultural stress profiles and study variables. Sensitivity analyses comparing the selected two-step approach with a single-step analysis using the AUXILIARY command in M*plus* indicated that results were nearly identical across both methods in terms of entropy and profile proportions, supporting the stability of the identified latent profiles. All final models were interpreted using relative risks and adjusted for sex, age, education, annual income, marital status, and birth region [46].

## Results

### Latent profile analysis

Table 1 provides the fit statistics for models ranging from two to five profiles. Findings supported the five-profile solution based on lower BIC, SSABIC, BLRT, and adjusted LMR-LRT values. The entropy fit statistic was the only indicator favoring the two-profile solution. After reviewing the statistical and substantive criteria and conducting a final visual assessment of the profiles across the two-, three-, four-, and five-profile models, we concluded that the five-profile solution best represented the data and offered statistical robustness and conceptual clarity.

**Table 1:** Fit statistics for latent profile analysis.

Fit statistics	2 Profiles	3 Profiles	4 Profiles	5 Profiles
**Latent profile model**				
**BIC**	49300.7	47405.0	46655.3	46177.8
**SSABIC**	49173.6	47233.5	46439.3	45917.3
**Entropy**	.938	.921	.887	.879
**Adj. LMR-LRT (*p-value*)**	6895.7 (<.001)	1977.0 (<.001)	842.3 (<.001)	572.7 (.004)
**BLRT (*p-value*)**	6964.5 (<.001)	1996.6 (<.001)	850.6 (<.001)	578.4 (<.001)
**Group size (n, %)**				
** Profile 1**	719, 53.2%	580, 42.9%	351, 26.0%	300, 22.2%
** Profile 2**	632, 46.8%	569, 42.1%	407, 30.1%	200, 14.8%
** Profile 3**		202, 15.0%	455, 33.7%	253, 18.7%
** Profile 4**			138, 10.2%	452, 33.5%
** Profile 5**				146, 10.8%

*Note.* BIC = Bayesian Information Criteria. SSABIC = Sample size adjusted BIC. Adj. LMR-LRT = Sample size adjusted Lo-Mendell-Rubin likelihood ratio test. BLRT = Bootstrap likelihood ratio test. *n* = profile sample size.

**Fig 1:**
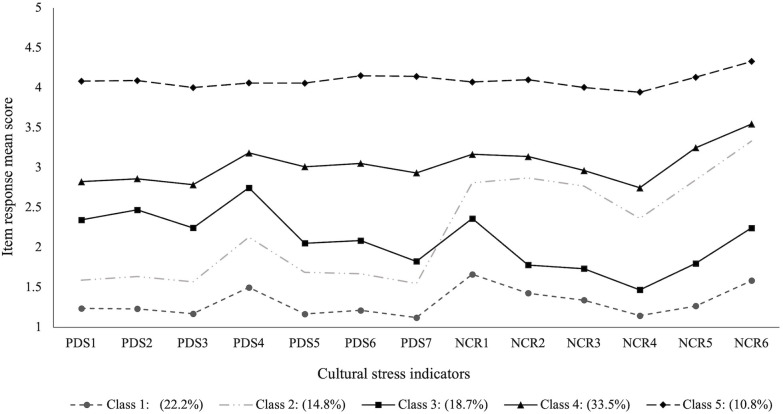
Latent profile analysis on cultural stress among Latin American immigrant parents in the US. *Note.* PDS = perceived discrimination. NCR = negative context of reception.

The interpretability of the five-profile solution model was evaluated by plotting the mean Likert-type response options (from *Strongly Disagree* to *Strongly Agree*) for the two cultural stress measures. For labeling, response options were used to create cultural stress thresholds. As shown in Fig. 1, we define such thresholds as “low,” “moderate,” “elevated,” and “highest.” We named each profile in the five-profile solution based on these thresholds and their combinations. Profile 1 was characterized by having *low levels of perceived discrimination and negative context of reception* (22.2%); Profile 2 included those individuals with *low levels of perceived discrimination and elevated negative context of reception* (14.8%); Profile 3 presented a *moderate level of both perceived discrimination and negative context of reception* (18.7%). Profile 4 was characterized by *elevated perceived discrimination and negative context of reception* levels (33.5%). Finally, Profile 5 included participants with *highest levels of both perceived discrimination and negative context of reception* (10.8%). In summary, these five profiles categorize our sample of parents based on their levels of cultural stressors–and their combinations–ranging from low to high among study participants.

### Latent profile characterization

Table 2 provides the distributions of sociodemographic characteristics across the five cultural stress profiles. No significant differences were found in terms of partnership status. However, we found significant differences in sex, age, birth region, post-tax household income, educational attainment, and nativity (all *p* values < .05).

**Table 2:** Sociodemographic characteristics and mental health outcomes across the four latent profiles.

	Total sample	Profile 1	Profile 2	Profile 3	Profile 4	Profile 5	*p-value*
**Sample Size**	1351	300	200	253	452	146	–
	(100%)	(22.2%)	(14.8%)	(18.7%)	(33.5%)	(10.8%)	
**Sociodemographic Characteristics**							
**Sex**							
** Male**	510 (38%)	91 (30%)	64 (32%)	87 (34%)	196 (43%)	72 (50%)	<.001
** Female**	836 (62%)	208 (70%)	135 (68%)	166 (66%)	255 (57%)	72 (50%)	
**Age (*M, SD*)**	39.8 (7.1)	41.1 (7.3)	39.3 (7.1)	40.4 (7.2)	39.4 (6.8)	38.00 (7.0)	<.001
**Education**							
** Less than college**	763(57%)	153 (51%)	124 (62%)	126 (50%)	276 (61%)	84 (58%)	.005
** College or more**	586 (43%)	146 (49%)	76 (38%)	127 (50%)	175 (39%)	62 (42%)	
**Household Income**							
** Less than $2000**	706 (52%)	132 (44%)	104 (52%)	112 (44%)	265 (59%)	93 (64%)	<.001
** $2000 or more**	645 (48%)	168 (56%)	96 (48%)	141 (56%)	187 (41%)	53 (36%)	
**Partnered**							
** Yes**	1022 (76%)	233 (78%)	149 (74%)	185 (73%)	346 (77%)	109 (75%)	.743
**Birth region**							
** North America (USA)**	511 (39%)	93 (32%)	83 (43%)	65 (27%)	208 (47%)	62 (43%)	<.001
** North America (Mexico)**	287 (22%)	53 (17%)	53 (27%)	37 (15%)	94 (21%)	50 (35%)	
** South America**	253 (19%)	75 (26%)	26 (13%)	70 (28%)	64 (14%)	18 (12%)	
** Central America**	98 (7%)	21 (7%)	8 (4%)	25 (10%)	37 (8%)	7 (5%)	
** Caribbean**	177 (13%)	52 (18%)	26 (13%)	49 (20%)	42 (9%)	8 (6%)	
**Immigration status**							
** First-generation**	840 (62%)	207 (69%)	117 (59%)	188 (74%)	244 (54%)	84 (58%)	<.001
** Second-generation**	586 (38%)	93 (31%)	83 (41%)	65 (26%)	208 (46%)	62 (42%)	
**Family functioning**							
** Family intimacy (*M, SD*)**	61.9 (9.7)	65.2 (7.5)	62.8 (8.0)	63.5 (8.9)	58.9 (10.9)	59.8 (10.2)	<.001
** Democratic parenting style (*M, SD*)**	38.1 (6.4)	38.9 (6.3)	38.5 (5.6)	38.6 (5.9)	36.9 (6.6)	38.5 (7.4)	<.001
** Family conflict (*M, SD*)**	31.2 (11.3)	26.2 (9.7)	30.3 (10.1)	28.3 (10.3)	33.2 (10.2)	41.8 (12.2)	<.001
**Behavioral and mental health outcomes**							
** Hazardous alcohol use (*M, SD*)**	7.2 (8.4)	3.6 (4.2)	5.9 (6.5)	4.9 (5.6)	8.5 (8.8)	16.4 (11.6)	<.001
** Depressive symptoms (*M, SD*)**	9.3 (6.7)	5.7 (5.2)	7.8 (5.9)	7.5 (5.7)	10.9 (6.1)	16.5 (6.7)	<.001
** Anxiety symptoms (*M, SD*)**	6.2 (6.0)	3.4 (4.7)	4.8 (5.1)	4.4 (5.2)	7.8 (5.7)	12.2 (6.4)	<.001

*Note.* Profile 1 = Low Perceived Discrimination Scale/Negative Context of Reception Scale. Profile 2 = Low Perceived Discrimination Scale/Elevated Negative Context of Reception Scale. Profile 3 = Moderate Perceived Discrimination Scale/Negative Context of Reception Scale. Profile 4 = Elevated Perceived Discrimination Scale/Negative Context of Reception Scale. Profile 5 = Highest Perceived Discrimination Scale/Negative Context of Reception Scale.

Regarding sex, Profile 5 (High discrimination/High negative context of reception) had the highest representation of men (50%). In comparison, Profile 1 (Low discrimination/Low negative context of reception) had a greater representation of women (70%). The youngest individuals were in Profile 5 (*M* = 38.0 years; *SD* = 7.0), and the oldest were in Profile 1 (*M* = 41.1 years; *SD* = 7.3).

In terms of participant birth region, North American parents born in the US were predominantly classified in Profile 4 (Elevated discrimination/Elevated negative context of reception), with 47% of parents falling into this category. In contrast, North American parents born in Mexico were most commonly placed into Profile 5 (Highest discrimination/Highest negative context of reception), accounting for 34%, indicating an even stronger perception of discrimination and negative context of reception. South American-born parents (28%) and those from Central America or the Caribbean (30%) were primarily categorized into Profile 3 (Moderate discrimination/Moderate negative context of reception). First-generation immigrant parents were most strongly represented within Profile 3 (Moderate discrimination/Moderate negative context of reception) with 74%, followed by Profile 1 (Low discrimination/Low negative context of reception), with 69%. Second-generation (US-born) immigrant parents were most strongly represented within Profile 4 (Elevated discrimination/Elevated negative context of reception) with 46%, followed by Profile 5 (Highest discrimination/Highest negative context of reception), with 42% (see Table 2). Profile 2 (Low discrimination/Elevated negative context of reception) was somewhat similar to Profile 5 in terms of nativity (~60% first generation).

Regarding family functioning, all three subscales (i.e., family intimacy, democratic parenting style, and family conflict) differed significantly across profiles. For example, participants reporting low levels of discrimination and negative context of reception (i.e., Profile 1) reported the highest levels of family intimacy (*M* = 65.2; *SD* = 7.5). In contrast, parents who reported the highest levels of discrimination and negative context of reception (i.e., Profile 5) reported lower family intimacy levels (*M* = 58.9; *SD* = 10.2). Participants who reported elevated perceived discrimination and negative context of reception (Profile 4) reported the lowest mean score for democratic parenting style (*M* = 36.9; *SD* = 6.6). Individuals in Profile 5, characterized by the highest levels of both cultural stressors, reported the highest levels of family conflict (*M* = 41.8; *SD* = 12.2).

When examining behavioral and mental health outcomes, significant differences emerged in hazardous alcohol use and in depressive and anxiety symptoms. Overall, parents who experienced the highest perceived discrimination and negative context of reception (i.e., Profile 5), reported greater mean scores of hazardous alcohol use (*M* = 16.4; *SD* = 11.6), depressive symptoms (*M* = 16.5; *SD* = 6.7), and anxiety symptoms (*M* = 12.2; *SD* = 6.4), relative to the other profiles.

### Multinomial logistic regression findings

Table 3 presents the association between profile membership and family functioning (i.e., family intimacy, democratic parenting style, and family conflict), hazardous alcohol use, and mental health outcomes (i.e., depressive and anxiety symptoms). When comparing parents reporting low levels of perceived discrimination and negative context of reception (i.e., Profile 1) to those with low levels of perceived discrimination but elevated negative context of reception (i.e., Profile 2), members of Profile 2 reported significantly less family intimacy (RRR = 0.97, 95% CI [0.95, 0.99]).

**Table 3:** Multinomial logistic regression model results.

	Profile 2 vs. Profile 1		Profile 3 vs. Profile 1		Profile 4 vs. Profile 1		Profile 5 vs. Profile 1	
	RRR	95% CI	RRR	95% CI	RRR	95% CI	RRR	95% CI
**Family intimacy**	0.97^*^	[0.95, 0.99]	0.97^*^	[0.94, 0.99]	0.93^*^	[0.91, 0.95]	0.94^*^	[0.92, 0.97]
**Democratic parenting style**	1.00	[0.97, 1.04]	0.99	[0.96, 1.02]	0.97^*^	[0.94, 0.99]	1.00	[0.97, 1.04]
**Family conflict**	1.03^*^	[1.01, 1.05]	1.02^*^	[1.01, 1.04]	1.06^*^	[1.04, 1.07]	1.13^*^	[1.10, 1.16]
**Hazardous alcohol use**	1.05^*^	[1.02, 1.09]	1.05^*^	[1.02, 1.09]	1.10^*^	[1.07, 1.14]	1.20^*^	[1.15, 1.24]
**Depressive symptoms**	1.07^*^	[1.03, 1.11]	1.07^*^	[1.03, 1.11]	1.16^*^	[1.12, 1.20]	1.31^*^	[1.26, 1.37]
**Anxiety symptoms**	1.05^*^	[1.01, 1.10]	1.05^*^	[1.01, 1.09]	1.16^*^	[1.12, 1.20]	1.29^*^	[1.24, 1.35]
**Elevated depressive symptoms**	2.07^*^	[1.34, 3.18]	1.57^*^	[1.02, 2.40]	4.40^*^	[3.06, 6.34]	22.94^*^	[12.61, 41.75]
**Elevated anxiety symptoms**	1.30	[0.74, 2.30]	1.37	[0.79, 2.38]	4.76^*^	[3.06, 7.41]	17.48^*^	[10.04, 30.41]

*Note.* CI = confidence interval. RRR = relative risk ratios. Profile 1 = Low Perceive Discrimination Scale/Negative Context of Reception Scale. Profile 2 = Low Perceive Discrimination Scale/Elevated Negative Context of Reception Scale. Profile 3 = Moderate Perceive Discrimination Scale/Negative Context of Reception Scale. Profile 4 = Elevated Perceive Discrimination Scale/Negative Context of Reception Scale. Profile 5 = Highest Perceive Discrimination Scale/Negative Context of Reception Scale. ^a^CES-D-10 score ≥ 10. ^b^GAD-7 score ≥ 10.

* = *p* <.05 or lower

Additionally, relative to Profile 1, parents in Profile 2 were more likely to report greater family conflict (RRR = 1.03, 95% CI [1.01, 1.05]), hazardous alcohol use (RRR = 1.05, 95% CI [1.02, 1.09]), depressive symptoms (RRR = 1.07, 95% CI [1.03, 1.11]), and anxiety symptoms (RRR = 1.05, 95% CI [1.01, 1.10]). Further, parents in Profile 2 were twice as likely to report clinically elevated depressive symptomatology (RRR = 2.07, 95% CI [1.34, 3.18]). No significant differences were observed for democratic parenting style or for elevated anxiety symptoms. A similar pattern was found when comparing Profile 1 to Profile 3 parents.

When comparing parents who reported low levels of perceived discrimination and a negative context of reception (i.e., Profile 1) to those who experienced both elevated levels of perceived discrimination and a negative context of reception (i.e., Profile 4), parents in Profile 4 reported lower levels of family intimacy (RRR = 0.93, 95% CI [0.91, 0.95]) and democratic parenting style (RRR = 0.97, 95% CI [0.94, 0.99]). Additionally, parents in Profile 4 were significantly more likely to experience family conflict (RRR = 1.06, 95% CI [1.04, 1.07]), hazardous alcohol use (RRR = 1.10, 95% CI [1.07, 1.14]), depressive symptoms (RRR = 1.16, 95% CI [1.12, 1.20]), and anxiety symptoms (RRR = 1.16, 95% CI [1.12, 1.20]). Furthermore, parents in Profile 4 were 4.40 times more likely to report clinically elevated depressive symptomatology (RRR = 4.40, 95% CI [3.06, 6.34]), and nearly five times more likely to report clinically elevated anxiety symptomatology (RRR = 4.76, 95% CI [3.06, 7.41]), compared to parents in Profile 1.

In comparison to Profile 1 (Low discrimination/Low negative context of reception), Latin American parents in Profile 5 (Highest discrimination/Highest negative context of reception) were 6% less likely to report family intimacy (RRR = 0.94, 95% CI [0.92, 0.97]). Moreover, parents in Profile 5 were significantly more likely to report family conflict (RRR = 1.13, 95% CI [1.10, 1.16]), hazardous alcohol use (RRR = 1.20, 95% CI [1.15, 1.24]), depressive symptoms (RRR = 1.31, 95% CI [1.26, 1.37]), and anxiety symptoms (RRR = 1.29, 95% CI [1.24, 1.35]). Notably, these parents were nearly 23 times more likely to report clinically elevated depressive symptomatology (RRR = 22.94, 95% CI [12.61, 41.75]) and 17.5 times more likely to report clinically elevated anxiety symptomatology (RRR = 17.48, 95% CI [10.04, 30.41]). We did not observe any significant difference in democratic parenting style in this comparison (i.e., Profile 5 vs Profile 1). Interestingly, no significant differences emerged when comparing parent in Profile 2 (Low discrimination/Elevated negative context of reception) with those in Profile 3 (Moderate discrimination/Moderate negative context of reception) across all independent variables studied (i.e., family functioning, hazardous alcohol use, and mental health outcomes; see S1 Table).

As shown in S1 Table, comparisons involving profiles characterized by elevated to high negative context of reception generally yielded similar results. Specifically, comparing Profile 2 (Low discrimination/Elevated negative context of reception) against Profile 4 (Elevated discrimination/Elevated negative context of reception) or Profile 5 (Highest discrimination/Highest negative context of reception) provided similar results as when Profile 1 (Low discrimination/Low negative context of reception) was the reference profile. Similar association patterns were found when comparing Profile 3 (Moderate discrimination/Moderate negative context of reception) participants against those in Profile 4 and/or Profile 5. In all comparisons, parents with greater cultural stress levels (i.e., Profile 4 and Profile 5) were more likely to report lower family intimacy levels. In contrast, Profile 4 and Profile 5 parents (i.e., elevated to high cultural stress) were more likely to report (1) greater levels of family conflict, hazardous alcohol use, depressive symptoms, and anxiety symptoms, and (2) elevated depressive and anxiety symptomatology. This pattern also held true when comparing members of Profile 4 to those in Profile 5 (Elevated vs. Highest), with one exception – democratic parenting style – that we outline in the next paragraph.

Finally, democratic parenting style exhibited mixed results, with (1) parents with elevated levels of both cultural stressors (i.e., Profile 4) having lower chances of providing greater democratic parenting style scores when compared with parents experiencing low discrimination but elevated negative context of reception (i.e., Profile 2; RRR = 0.96, 95% CI [0.94, 0.99]), but (2) parents with high levels of both cultural stressors (i.e., Profile 5) associated with greater odds of reporting greater democratic parenting style compared to parents with elevated cultural stress levels (i.e., Profile 4; RRR = 1.03, 95% CI [1.01, 1.07]). The remaining results for democratic parenting style were not statistically different across profiles.

## Discussion

The present study was designed to characterize underlying cultural stress profiles and to examine their association with family functioning, hazardous alcohol use, and mental health symptoms among distinct subgroups of immigrant Latin American parents residing in the United States. Five different profiles or subgroups of parents were identified based on their levels of cultural stress. Unique sociodemographic factors, including sex, age, birth region, income, education, and immigration status, characterized each latent subgroup. Significant differences emerged among these profiles concerning family intimacy, democratic parenting, family conflict, hazardous alcohol use, and mental health outcomes.

Our identified profile solution aligns partially with previous research conducted among different populations (e.g., non-immigrant Latin American populations such as Puerto Ricans [47]. For instance, Piñeros-Leaño et al. [48] identified distinct subgroups of cultural stress (e.g., overall low cultural stress, moderate cultural stress, high cultural stress, and low language-related stress) among Hurricane Maria survivors from Puerto Rico—a group of Latin American individuals who, although experiencing cultural stress, is not considered an immigrant population in the United States. Additionally, that study incorporated language-related stress as a key dimension of cultural stress. Nonetheless, our results are at least somewhat similar to those from that previous study, in the sense that we identified subgroups characterized by varying levels of one or more cultural stressors. However, in contrast to Piñeros-Leaño et al. [48], we found a profile that was low in discrimination but high in negative context of reception – a profile in which U.S.-born participants were overrepresented. (No participants in Piñeros-Leaño et al. were born on the U.S. mainland.) Our findings underscore the importance of taking into account contextual and population-specific factors when conceptualizing and identifying cultural stress subgroups. Different Latin American populations may experience cultural stressors differently, particularly given the distinct sociopolitical challenges faced by immigrants (e.g., immigration stress and the threat of deportation) compared to U.S. citizens.

### Cultural stress profiles characteristics

We identified some notable associations and differences between latent cultural stress profiles and sociodemographic characteristics. To begin, the gender distribution was significantly different across cultural stress profiles, with greater proportions of men found in profiles characterized by elevated and highest discrimination and the negative context of reception. This finding supports prior research that suggests men are likely to experience more discrimination than women are [49].

We found that the majority of South American and Caribbean parents in our sample were classified into the low and moderate cultural stress profiles. In contrast, North American Mexican-origin participants were more likely to be placed into the elevated and high cultural stress groups. This finding aligns with research by Montero-Zamora et al. [28], which found that both discrimination and negative context of reception were significantly greater among first-generation Mexican parents in the U.S. compared to parents from other Latin American countries. These results suggest that Mexican-origin immigrant parents may encounter unique cultural stressors that heighten their vulnerability to family dysfunction, alcohol-related issues, anxiety, and depressive symptoms. This disparity may be attributed to structural, historical, or sociopolitical factors uniquely shaping the migration experiences of different Latin American subgroups, with Mexicans being the most represented group given its role as a border country with the U.S. [50]. Additionally, many U.S. residents maintain negative stereotypes about Mexican immigrants [51]– potentially contributing to greater cultural stress for parents born in Mexico.

Furthermore, our findings suggest the existence of a distinct subgroup marked by low discrimination but elevated levels of negative context of reception. A key sociodemographic trait differentiating this subgroup from the low and moderate cultural stress profiles is the greater proportion of U.S.-born or second-generation individuals. This result suggests that, despite facing lower levels of discrimination–perhaps due to greater English language proficiency–second-generation Latin American individuals may still confront significant contextual stressors that influence their experiences in U.S. society [52]. Meca et al. [53] found that, among a sample of Latin American descent emerging adults, U.S.-born individuals were less likely than their foreign-born counterparts to belong to a profile characterized by high negative context of reception and low discrimination (rather than to a profile with low levels of both cultural stressors). However, these differences were also linked with heritage-cultural rejection—an additional indicator included in their latent profile analysis. Heritage rejection was defined by Meca et al. [53] as an acculturative strategy characterized by low engagement in Hispanic cultural practices and by low ethnic affirmation. These constructs, however, were not included in our latent profile analysis, which specifically focused on cultural stress rather than its interaction with other factors, such as acculturative strategies. Nonetheless, our findings appear similar to those reported by Meca et al.

### Cultural stress and family functioning

In terms of family relationship variables, we found that greater levels of cultural stress were associated with decreased family intimacy and increased family conflict. Consistent with our findings, prior studies with Latin American families in the United States suggest that cultural stress may influence family relationships by heightening overall stress and increasing the likelihood of family conflict [23,54]. According to family systems theory [55], human functioning is shaped by interactions within and between the family and its broader context. When cultural stressors influence that context, these interactions might be disrupted, perhaps leading to family dysfunction [56].

Additionally, the democratic parenting style appeared to have mixed associations with the cultural stress profiles. This pattern may be attributed to (1) unobserved sociodemographic characteristics within the sample that were not included in our study (e.g., year of arrival to the U.S. among foreign-born participants) or (2) heterogeneity in the development of family functioning over time, as indicated in previous studies [22]. Our results highlight the need to (1) account for the duration of time spent in the destination country in future studies and (2) integrate family dynamics into interventions to mitigate the adverse effects of cultural stress. For instance, programs such as *Familias Unidas* [57] or *Guiding Good Choices* [58] could incorporate into their curricula strategies for helping Latin American descent families more effectively navigate cultural stress in the United States – where these strategies would likely differ based on the parents’ nativity status and time in living in the United States. Given that families are an important source of stability and support for immigrants [19], understanding the potential impact of cultural stress on family dynamics is crucial for guiding future research.

### Cultural stress and hazardous alcohol use

Regarding behavioral outcomes, the findings indicate that parents experiencing greater levels of cultural stress were more likely to engage in hazardous alcohol use. This finding is consistent with existing research, which has suggested a positive association between harmful alcohol consumption and perceived discrimination among Latin American immigrants in the United States. For example, Macias Burgos et al. [59] found that discrimination was significantly linked with hazardous alcohol use in a sample of Latin American immigrants in the U.S., even after controlling for depressive and anxiety symptoms. Additionally, the association between cultural stress and alcohol use, particularly among parents like those in our sample, may be explained by the disruptive link between cultural stress and family functioning. This disruption, in turn, could increase the likelihood of alcohol abuse among family members among first-generation Latin American immigrant parents [14,28].

### Cultural stress and mental health

Our findings regarding mental health outcomes suggested a pattern similar to that observed for hazardous alcohol use. Specifically, individuals classified into higher cultural stress profiles were more likely than members of other classes to report elevated depressive symptoms. A similar trend was observed for anxiety symptoms. These results are consistent with previous research suggesting a possible association between perceived discrimination, the negative context of reception, depressive [16,25,36,60] and anxiety [61–63] symptoms among Latin American families.

Furthermore, our findings suggested that individuals experiencing elevated (i.e., Profile 4) and highest (i.e., Profile 5) levels of cultural stress may be more likely to report elevated of depressive and anxiety symptomatology. This increase in the likelihood of mental health symptoms with higher cultural stress levels underscores the need for targeted interventions, as those with high cultural stress levels may benefit from indicated prevention strategies for mental health concerns [64]. Regarding the mechanisms underlying these associations, adverse mental health outcomes among Latin American families resulting from greater cultural stress may be explained by impairments in adaptive coping mechanisms, which, in turn, might have been initially triggered by prolonged exposure to cultural stressors [65].

Overall, our findings emphasize the need for culturally tailored interventions across the full spectrum of prevention (i.e., universal, selective, and indicated) to effectively address both cultural stress and mental health challenges within Latin American descent families in the United States. For example, universal prevention programs could focus on mitigating cultural stress tensions between parents and children, particularly by enhancing family functioning. Selective and indicated prevention programs could provide specialized support for specific family members (e.g., mothers, fathers, or children) at high risk or already experiencing elevated depressive and anxiety symptoms related to moderated or high cultural stress levels (e.g., cognitive behavioral therapy strategies [66]).

### Limitations and future directions

The present findings should be interpreted in light of several important limitations. First, the cross-sectional design limits the ability to infer causality and ascertain the temporal relationship between cultural stress profiles and outcomes such as family functioning, hazardous alcohol use, and symptoms of depression and anxiety. Future longitudinal research could provide deeper insight into these relationships by establishing temporal sequencing and causality. Second, the measurement of cultural stress used to define the latent subgroups was limited to two specific stressors (i.e., perceived discrimination, negative context of reception). However, cultural stress is integrated by multiple constructs, such as language-related and bicultural stress [67]. Future studies should incorporate these and other additional constructs to determine whether the observed patterns of associations remain consistent.

Third, among first-generation individuals in our sample, information regarding participants’ migration characteristics was limited to their country or region of origin. Potentially relevant push (e.g., war, conflict, or natural disasters) and pull (e.g., employment or educational opportunities) factors underlying migration were not measured or incorporated into our analyses. Including such variables in future research would provide a more comprehensive understanding of how pre-migration and migration-related experiences may shape cultural stress subgroups among Latin American families. In line with the importance of considering contextual factors, we also acknowledge that geospatial and neighborhood characteristics of participants’ residential environments (e.g., residing in a Latino enclave) were not available for analysis. Future research should investigate whether the number and features of cultural stress profiles vary across different geographical or spatial contexts within the United States.

Fourth, all data were collected through self-report measures, which may introduce social desirability bias. Incorporating objective measures of the outcomes in future research could enhance the reliability and validity of the findings. Five, this study used a non-probabilistic sample (i.e., purposive) of Latin American parents in the U.S. with a restricted range of child ages, limiting the generalizability of results to the broader Latin American population. Future studies should aim to include more diverse samples and consider probabilistic sampling methods to improve the applicability of findings across different subgroups within the Latin American community.

Despite these and other limitations, our study fills a gap in the existing literature by empirically identifying distinct cultural stress profiles related to perceived discrimination and negative contexts of reception. Specifically, our study contributes to the empirical knowledge base by (1) utilizing widely recognized and established scales among Latino immigrant parents in the U.S., (2) examining the role of different regions of origin and their association with these stress profiles, (3) expanding the body of evidence on differences between first- and second-generation Latin American individuals in the U.S., and (4) providing valuable insights to inform prevention and implementation science, particularly in developing and adapting targeted interventions for Latin American populations.

## Conclusions

Our findings suggest the presence of especially vulnerable subgroups due to cultural stress among Latin American parents in the United States. Cultural stress plays a critical role as a social determinant of health, being differentially associated with the well-being of Latin American immigrant families in the United States. Greater cultural stress is associated with negative family dynamics, increased hazardous alcohol use, and poorer mental health. These findings can guide future research aimed at developing theoretical frameworks to better understand how cultural stress interacts with potential sources of resilience (e.g., family intimacy) in shaping behavioral and mental health outcomes among Latino families in the U.S.

Importantly, our results underscore the need for culturally tailored interventions across the full spectrum of prevention, including universal strategies to strengthen family functioning and selective or indicated approaches targeting high-risk parents and families. Practitioners and policymakers can use these findings to identify families at elevated risk and implement evidence-based programs that account for nativity and specific stressors experienced by different Latin American subgroups. Furthermore, future research should incorporate additional dimensions of cultural stress (e.g., language-related stress, bicultural stress) and employ longitudinal designs to clarify causal pathways and inform more effective interventions.

A better understanding of cultural stress patterns may improve both current and future theoretical frameworks and evidence-based interventions tailored for Latin American families, addressing health disparities within this population. We hope that the present study inspires additional work in this direction.

## Supporting information

S1 Table Multinomial logistic regression model results. *Note.* CI = confidence interval. RRR = relative risk ratios. Profile 1 = Low Perceive Discrimination Scale/Negative Context of Reception Scale. Profile 2 = Low Perceive Discrimination Scale/Elevated Negative Context of Reception Scale. Profile 3 = Moderate Perceive Discrimination Scale/Negative Context of Reception Scale. Profile 4 = Elevated Perceive Discrimination Scale/Negative Context of Reception Scale. Profile 5 = Highest Perceive Discrimination Scale/Negative Context of Reception Scale. ^a^CES-D-10 score ≥ 10. ^b^GAD-7 score ≥ 10. ^*^ = *p *< .05 or lower.
(DOCX)

S1 Data (ZIP)
