# Supplementary material for: Cultural stress, family functioning, hazardous alcohol use, and mental health among Latin American parents in the United States: A latent profile analysis
Source: PLoS One. 2025 Nov 21;20(11):e0337543. doi: 10.1371/journal.pone.0337543 (PMC12637974; doi:10.1371/journal.pone.0337543)
Supplement: S1 Table — Note. CI = confidence interval. RRR = relative risk ratios. Profile 1 = Low Perceive Discrimination Scale/Negative Context of Reception Scale. Profile 2 = Low Perceive Discrimination Scale/Elevated Negative Context of Reception Scale. Profile 3 = Moderate Perceive Discrimination Scale/Negative Context of Reception Scale. Profile 4 = Elevated Perceive Discrimination Scale/Negative Context of Reception Scale. Profile 5 = Highest Perceive Discrimination Scale/Negative Context of Reception Scale. aCES-D-10 score ≥ 10. bGAD-7 score ≥ 10. * = p < .05 or lower. (DOCX) [file pone.0337543.s001.docx]

|  | **Profile 3 vs. Profile 2** | | **Profile 4 vs. Profile 2** | | **Profile 5 vs. Profile 2** | | **Profile 4 vs. Profile 3** | | **Profile 5 vs. Profile 3** | | **Profile 5 vs. Profile 4** | |
| --- | --- | --- | --- | --- | --- | --- | --- | --- | --- | --- | --- | --- |
|  | RRR | 95% CI | RRR | 95% CI | RRR | 95% CI | RRR | 95% CI | RRR | 95% CI | RRR | 95% CI |
| **Family intimacy** | 1.00 | [0.97, 1.02] | 0.96^*^ | [0.94, 0.98] | 0.97^*^ | [0.95, 0.99] | 0.97^*^ | [0.95, 0.98] | 0.97^*^ | [0.95, 0.99] | 1.01 | [0.99, 1.03] |
| **Democratic parenting style** | 0.99 | [0.96, 1.02] | 0.96^*^ | [0.94, 0.99] | 1.00 | [0.96, 1.04] | 0.97 | [0.95, 1.00] | 1.01 | [0.97, 1.05] | 1.03^*^ | [1.01, 1.07] |
| **Family conflict** | 0.99 | [0.97, 1.1] | 1.02^*^ | [1.00, 1.04] | 1.09^*^ | [1.06, 1.12] | 1.03^*^ | [1.01, 1.05] | 1.10^*^ | [1.08, 1.13] | 1.07^*^ | [1.05, 1.09] |
| **Hazardous alcohol use** | 1.00 | [.97, 1.03] | 1.04^*^ | [1.02, 1.07] | 1.13^*^ | [1.10, 1.17] | 1.05^*^ | [1.02, 1.07] | 1.14^*^ | [1.10, 1.17] | 1.09^*^ | [1.06, 1.11] |
| **Depressive symptoms** | 1.00 | [.97, 1.04] | 1.09^*^ | [1.05, 1.12] | 1.23^*^ | [1.18, 1.28] | 1.08^*^ | [1.05, 1.11] | 1.22^*^ | [1.18, 1.27] | 1.13^*^ | [1.09, 1.17] |
| **Anxiety symptoms** | 1.00 | [.96, 1.04] | 1.10^*^ | [1.05, 1.14] | 1.23^*^ | [1.18, 1.28] | 1.10^*^ | [1.07, 1.14] | 1.23^*^ | [1.18, 1.28] | 1.12^*^ | [1.08, 1.15] |
| **Elevated depressive symptoms** | 0.75 | [0.49, 1.17] | 2.13^*^ | [1.47, 3.07] | 11.09^*^ | [6.10, 20.16] | 2.81^*^ | [1.94, 4.05] | 14.61^*^ | [8.02, 26.62] | 5.21^*^ | [3.00, 9.03] |
| **Elevated anxiety symptoms** | 1.05 | [0.59, 1.87] | 3.66^*^ | [2.31, 5.81] | 13.43^*^ | [7.60, 23.73] | 3.47^*^ | [2.23, 5.41] | 12.73^*^ | [7.31, 22.16] | 3.66^*^ | [2.37, 5.68] |
